# ASC speck serum concentrations, a component of sterile cellular inflammation, are associated with individual cardiopulmonary capacity

**DOI:** 10.3389/fphys.2024.1394340

**Published:** 2024-10-01

**Authors:** Alexander Kogel, Nell Voßhage, Amirhossein Behzadi, Ulrich Laufs, Sven Fikenzer

**Affiliations:** Klinik und Poliklinik für Kardiologie, Universitätsklinikum Leipzig, Leipzig, Germany

**Keywords:** inflammasome, CPET, ASC specks, oxygen pulse, cardiopulmonary capacity

## Abstract

**Aims:**

Exercise-induced cellular stress and sterile inflammation are of increasing interest. ASC specks are a component of the intracellular NLRP3-inflammasome and can be released into the blood. For example, serum ASC specks are increased after marathon running. We therefore tested whether ASC specks are potentially associated with the individual response to physical training and cardiopulmonary capacity.

**Methods:**

We performed a prospective study in 45 healthy athletes. Blood samples were taken before and after cardiopulmonary exercise testing (CPET). ASC speck concentrations were quantitated using flow cytometry.

**Results:**

Baseline ASC speck levels correlated with clinical parameters of body composition (height, weight, BMI) and parameters of cardiopulmonary performance (peak VO2, peak oxygen pulse, heart rate after exercise). Athletes with lowest baseline ASC speck concentrations have a significantly lower BMI (22.0 ± 1.8 vs. 24.9 ± 1.6 kg/m^2^), higher heart rate at rest (72 ± 10 vs. 58 ± 10 beats/min), lower peak VO2 (2692 ± 629 vs. 3404 ± 747 mL/min) and lower peak oxygen pulse (15.6 ± 3.4 vs. 20.7 ± 3.5 mL/heart rate). Overall, ASC speck concentrations showed no significant change after CPET (7.0 ± 4.5 vs. 8.0 ± 5.4 ASC specks/µL, *p* = 0.3). However, subgroup analysis revealed a significant increase in circulating ASC specks in athletes with the lowest baseline values (2.37 ± 0.84 vs. 8.43 ± 7.52 ASC specks/µL, *p* < 0.05). Athletes with an increase in ASC speck concentrations in response to CPET had a lower peak oxygen pulse compared to those with a decrease (17.1 ± 4.2 vs. 19.8 ± 4.1, *p* < 0.05).

**Conclusion:**

Low ASC speck baseline values as well as an increase in response to exercise are associated with lower peak oxygen pulse in healthy athletes.

## Introduction

Low physical activity and a sedentary lifestyle are well known risk factors for morbidity and mortality (Global burden of 87 risk, 2020). Positive effects of regular physical activity include reduction of the risk of cardiovascular diseases or cancer (Kyu et al., 2016; Aune et al., 2015; Patel et al., 2019). In contrast to moderate endurance training, high volumes of high-intensity exercise bouts in marathon runners have been suggested to be associated with negative health effects such as coronary calcification and myocardial fibrosis (Mohlenkamp et al., 2008; Breuckmann et al., 2009). In general, the effect of additional physical activity is decreasing with higher volumes resulting in a J- or U-shaped dose-response curve of physical acitivty and mortality (Arem et al., 2015; Eijsvogels et al., 2018). The underlying mediators for potential negative effects induced by extreme exercise are incompletely understood. In addition, there is need for serum markers that may help to predict and guide individual response to physical training and to assess cardiopulmonary capacity.

Potential mediators induced by extreme exercise include mechanisms of sterile vascular inflammation. Several inflammatory markers such as interleukin-6, tumor necrosis factor-α, and high-sensitive C-reactive protein (hsCRP) have been shown to increase in response to extreme exercise (Bernecker et al., 2013; Scherr et al., 2011; Kaufmann et al., 2021). In a previous study, we observed a transient increase in circulating apoptosis associated speck-like protein containing a CARD (ASC) specks after performing a marathon run (Kogel et al., 2022). Circulating ASC specks are activated inflammasome particles which are released into circulation during pyroptosis, the inflammatory cell death induced by inflammasome activation. These particles may then perpetuate inflammation and can be internalized by smooth muscle cells or endothelial cells (Kogel et al., 2022; Franklin et al., 2014; Gaul et al., 2021). In cardiovascular disease, inflammasome activation is associated with progression of atherosclerosis (Paramel et al., 2016; Grebe et al., 2018).

While marathon is an extreme form of physical exhaustion with a long duration of high mechanical muscle strain, cardiopulmonary exercise testing (CPET) does also induce exhaustion but in a much shorter time. Short, high intensity exercise is known to be associated with several health benefits (Atakan et al., 2021).

Based on this we designed a prospective observational study to test the hypothesis that the response of circulating ASC specks to a short high intensity bout of exercise may be associated with the individual response to physical training and cardiopulmonary capacity.

## Methods

### Subjects

We enrolled and analyzed 45 cardiovascularly healthy athletes, defined as an individual of young or adult age, either amateur or professional, who is engaged in regular exercise training and participates in official sports competition in accordance to ESC guidelines (24 males, 21 females) at the outathlet clinic (Pelliccia et al., 2020). Eleven athletes were male participants of the highest division of male handball, seven were females in the second division of female handball, thirteen were participants in the fourth division of male football and fourteen were participants in the first division of female football. All CPET tests were performed during the pre-season. The study was conducted in accordance with the Declaration of Helsinki on Ethical Principles and was approved by the Ethical Committee of the Medical Faculty, University of Leipzig (reference number 050/23-ek). Informed consent was obtained from all participants.

#### Study design

We performed a prospective observational study. Subjects received a physical examination, body measurements, and a resting electrocardiogram (ECG). Each athlete was examined via transthoracic echocardiography. After excluding signs of acute illness, an incremental cardiopulmonary exercise testing (CPET) was performed. Respiratory function was quantified using spirometry. Blood samples were taken before (T0) and 15 min after CPET (T1).

#### Blood parameters and flow cytometric analysis of ASC specks

Hematocrit, hemoglobin, erythrocytes, thrombocytes, leukocytes, potassium, sodium, chloride, ferritin, creatinine, creatine kinase (CK), creatine kinase myocardial band (CK-MB), hsCRP, high sensitive troponin (hsTroponin), N-terminal-pro-brain natriuretic peptide (NT-proBNP) and ASC specks were measured by the central lab of Leipzig University Hospital. Due to technical issues, CK and CK-MB measurements for one participant were unavailable.

Serum ASC specks were quantitated by flow cytometry as described (Kogel et al., 2022). 75 μL of human serum was diluted in 225 µL phosphate-buffered saline and centrifuged at 17,000 × g for 20 min at 4°C to isolate microparticles. After centrifugation, the supernatant was discarded and the pellet was resuspended in 300 µL PBS. Samples were then stained using ASC-B3-Alexa-647 (sc-514414, Santa Cruz) for 1 h at 37°C. Afterwards, 120 µL of the samples were measured using a flow cytometer BDFacsLyric (BDscience). Intra- and interassay of ASC speck measurements were previously published (Kogel et al., 2022).

#### Spirometry

The spirometry data as vital capacity (VC), forced expiratory volume in 1 s (FEV1), Tiffenau and peak expiratory flow (PEF) were collected using using a digital spirometer (Vyntus™ CPX, Vyaire Germany, Hoechberg, Germany).

#### Cardiopulmonary exercise tests (CPET)

CPET was performed on a semi-recumbent ergometer (GE eBike, GE Healthcare GmbH, Solingen, Germany) at a constant speed of 60–70 revolutions per minute. The test began at a workload of 50 W with an increase of 50 W every 3 min until volitional exhaustion occurred. The achievement of a respiratory exchange ratio of ≥1.1 and the subjective perception of exertion of 10 on the modified Borg scale (0–10) was defined as a criterion for adequate volitional effort. Each subject continued for an additional 5-min recovery period at a workload of 25 W. In the CPET, spiroergometry data were collected using a digital spirometer (Vyntus™ CPX, Vyaire Germany, Hoechberg, Germany). Oxygen uptake (VO2), respiratory minute volume (VE), and heart rate (HR) (GE-Cardiosoft, GE Healthcare GmbH, Solingen, Germany) were monitored continuously at rest, during CPET, and during recovery.

The first and second ventilatory threshold (VT1 and VT2, respectively) were determined using the graphs proposed by Wasserman et al. (2004), numbers six and nine (ventilatory equivalents and final expiratory pressure of oxygen and carbon dioxide, respectively).

VT1 was defined as an increase in the VE/VO2 and end-tidal PO2 (PETO2) without a concomitant increase in VE/VCO2.

VT2 was determined when an increase in the VE/VO2 and VE/VCO2 and a decrease in end-tidal PCO2 (PETCO2) were observed.

#### Interleukin quantification

Interleukin concentrations were measured using commercially available kits according to the manufacturer’s protocol. For IL-1β: Human IL-1β/IL-1F2 DuoSet ELISA Kit by R&D systems, and Interleukin-18: Human Total IL-18 DuoSet ELISA Kit by R&D systems were used, respectively.

### Statistical analysis

Data were analyzed using Microsoft Office Excel 365 for Windows (Microsoft Corporation, Redmond, Washington, United States) and GraphPad Prism 10 (Version 10 GraphPad Software Inc, California, United States). The significance level was set at *p* < 0.05. Differences between two groups were analyzed by a two-sided Student’s t-test. Intraindividual changes were analyzed with paired Student’s t-test. Data are expressed as mean ± standard deviation (SD) if not stated otherwise. Correlation analysis was performed using JASP 0.18.2 (JASP Team, 2024). Shapiro-Wilk test was used to test for normality, if normally distributed Pearson’s correlation was used, if not Spearman’s correlation was used instead. As ASC speck measurements were not normally distributed all correlation analyses including ASC specks were calculated using Spearman’s rank correlation (rho (ρ). CorelDraw2020 was used to create the figures.

## Results

### Low ASC speck baseline values correlate to lower BMI as well as lower cardiopulmonary capacity

**FIGURE 1:**
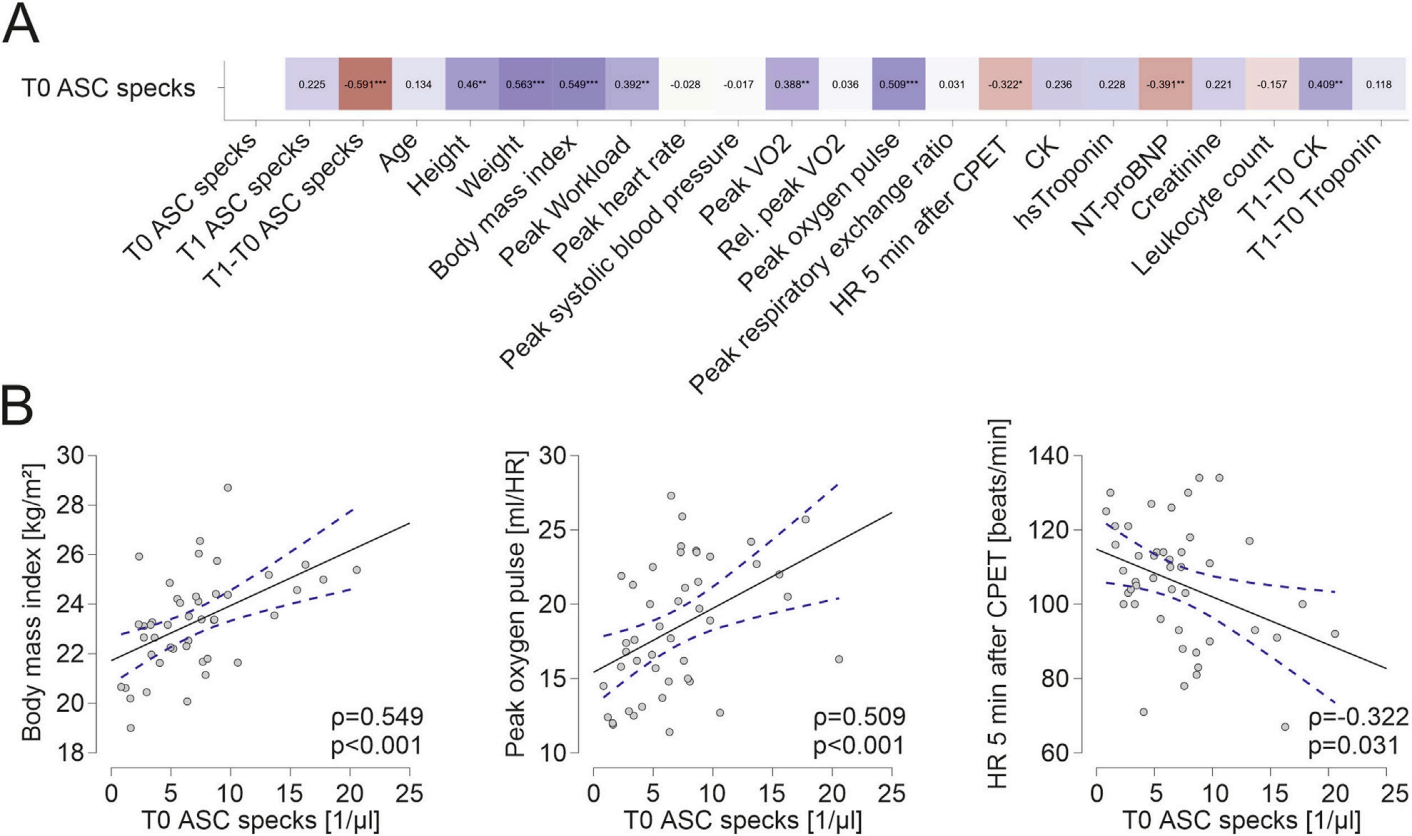
Extracellular ASC specks correlate to parameters of body composition and parameters of cardiopulmonary capacity **(A)** Spearman correlation matrix of baseline extracellular ASC specks with clinical parameters, parameters of CPET and blood parameters. **(B)** Exemplary scatter plots and linear regression (95% confidence interval in blue) of correlations of extracellular ASC specks. The Spearman correlation coefficient was used to analyze the data. *p < 0.05,**p < 0.01,***p < 0.001.

**FIGURE 2:**
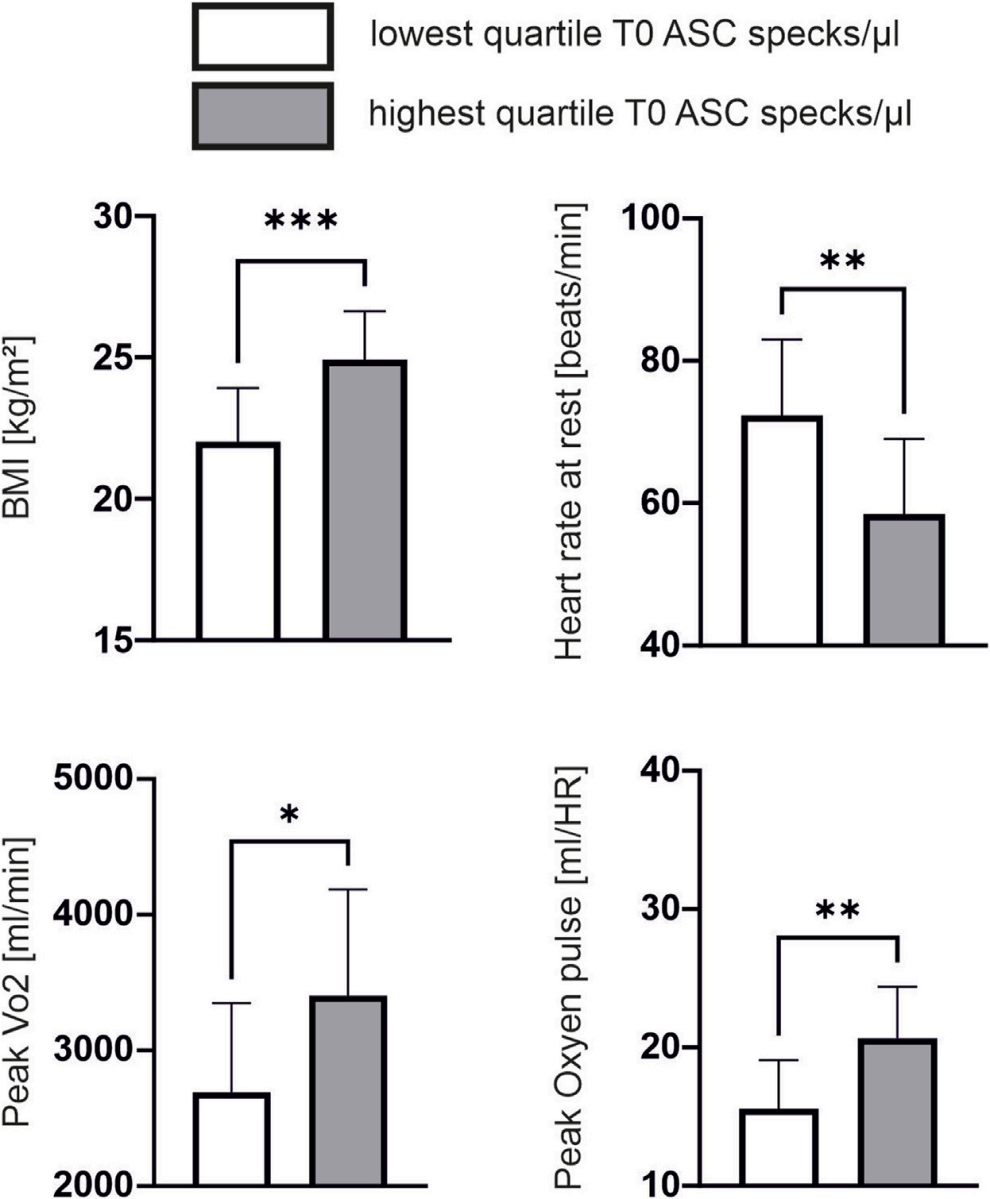
Lower cardiopulmonary performance is associated with lower ASC speck baseline values Subgroup comparison of the quartile with lowest ASC speck concentration at baseline (n = 12) compared to the quartile with highest ASC speck concentration at baseline (n = 11). Data are expressed as mean ± standard deviation *p < 0.05,**p < 0.01,***p < 0.001. Student-t tests were used to analyze the data.

Baseline characteristic of athletes are shown in Table 1. Baseline ASC speck concentrations showed a significant positive correlation to parameters of body composition such as height (ρ = 0.460, *p* = 0.001, n = 45), weight (ρ = 0.563, *p* < 0.001) and BMI (ρ = 0.549, *p* < 0.001; Figure 1A). Additionally, we found a significant positive correlation to parameters of cardiopulmonary capacity such as peak workload (ρ = 0.392, *p* = 0.008), peak VO2 (ρ = 0.388, *p* = 0.008) and peak oxygen pulse (ρ = 0.509, *p* < 0.001; Figure 1B). Heart rate 5 min after CPET as surrogate for fitness was also negatively correlated to baseline ASC specks (ρ = −0.322, *p* = 0.031; Figure 1B). At submaximal levels of exercise baseline ASC specks correlate significantly to VO2 (ρ = 0.376, *p* = 0.011) and relative heart rate (ρ = −0.338, *p* = 0.023) at VT1 as well as workload (ρ = 0.425, *p* = 0.004) and VO2 (ρ = 0.401, *p* = 0.006) at VT2. There was no significant correlation to cardiac serum parameters such an hsTroponin (n = 45), NT-proBNP (n = 45), CK (n = 44) or CK-MB (n = 44; Figure 1A). Comparing the quartile with lowest to the quartile with highest ASC baseline values, we found that the athletes in the lowest quartile had a significant lower BMI (22.0 ± 1.8 vs. 24.9 ± 1.6 kg/m^2^, *p* < 0.001), higher heart rate at rest (72 ± 10 vs. 58 ± 10 1/min, *p* = 0.003), lower peak VO2 (2692 ± 629 vs. 3404 ± 747 mL/min, *p* = 0.02) and lower peak oxygen pulse (15.6 ± 3.4 vs. 20.7 ± 3.5 mL/HR, *p* = 0.002; Figure 2).

**TABLE 1:** Characteristics of athletes.

Clinical Parameter	Mean (SD)
N (m; f)	45 (24; 21)
Age, y	23.4 (4.4)
Height, cm	179.1 (10.8)
Weight, kg	75.4 (13.6)
BMI, kg/m^2^	23.3 (2.0)
Blood parameter	
Hematoccrit, %	0.41 (0.03)
Heamoglobin, mmol/L	8.6 (0.8)
Erythrocytes, mmol/L	4.7 (0.4)
Thrombocytes, mmol/L	254.3 (38.7)
Leukocytes, mmol/L	6.3 (1.5)
Potassium, mmol/L	4.3 (0.4)
Sodium, mmol/L	139.8 (1.3)
Chloride, mmol/L	102.4 (1.8)
Ferritin, mmol/L	75.7 (59.4)
C-reactive proteine, mg/L	1.5 (1.8)
Echocardiographic measurements	
LVEF, %	64.8 (5.6)
Spirometry	
VC, l	5.3 (1.1)
FEV1, L/s	4.4 (0.9)
Tiffenau, %	84.5 (6.2)
PEF, L/s	9.1 (1.9)
CPET	
VT1	
Workload, Watt	113.5 (28.9)
Heart rate, beats/min	118.6 (16.1)
VO2, mL/min	1512 (355)
Rel. VO2, mL/min*kg	20.1 (3.2)
Oxygen pulse, mL/HR	13.0 (3.4)
VT2	
Workload, Watt	169.5 (48.4)
Heart rate, beats/min	155.2 (17.2)
VO2, mL/min	2390 (633)
Rel. VO2, mL/min*kg	31.6 (5.7)
Oxygen pulse, mL/HR	15.5 (4.1)
Peak	
Workload, Watt	235.9 (53.8)
Heart rate, beats/min	182.5 (11.4)
Systolic blood pressure, mmHg	207.7 (26.1)
Diastolic blood pressure, mmHg	86.7 (13.4)
VO2, mL/min	3123 (747)
Rel. VO2, mL/min*kg	41.3 (5.9)
VE, L/min	111.5 (27.6)
Oxygen-pulse, mL/HR	18.4 (4.4)
Peak respiratory exchange ratio	1.13 (0.06)
5 min after CPET	
Heart rate, beats/min	105.8 (16.3)

N, numbers; m, male; f, female; y, years; LVEF, left ventricular ejection fraction; VC, vital capacity, FEV1, forced minute volume in 1s, PEF, peak expiratory flow; HR, heart rate; VO2, oxygen uptake, VE, respiratory minute volume; VT1, first ventilatory threshold; VT2, second ventilatory threshold.

### Performing a CPET results in an increase in ASC speck serum concentrations only in cardiovascularly healthy athletes with low baseline values

**FIGURE 3:**
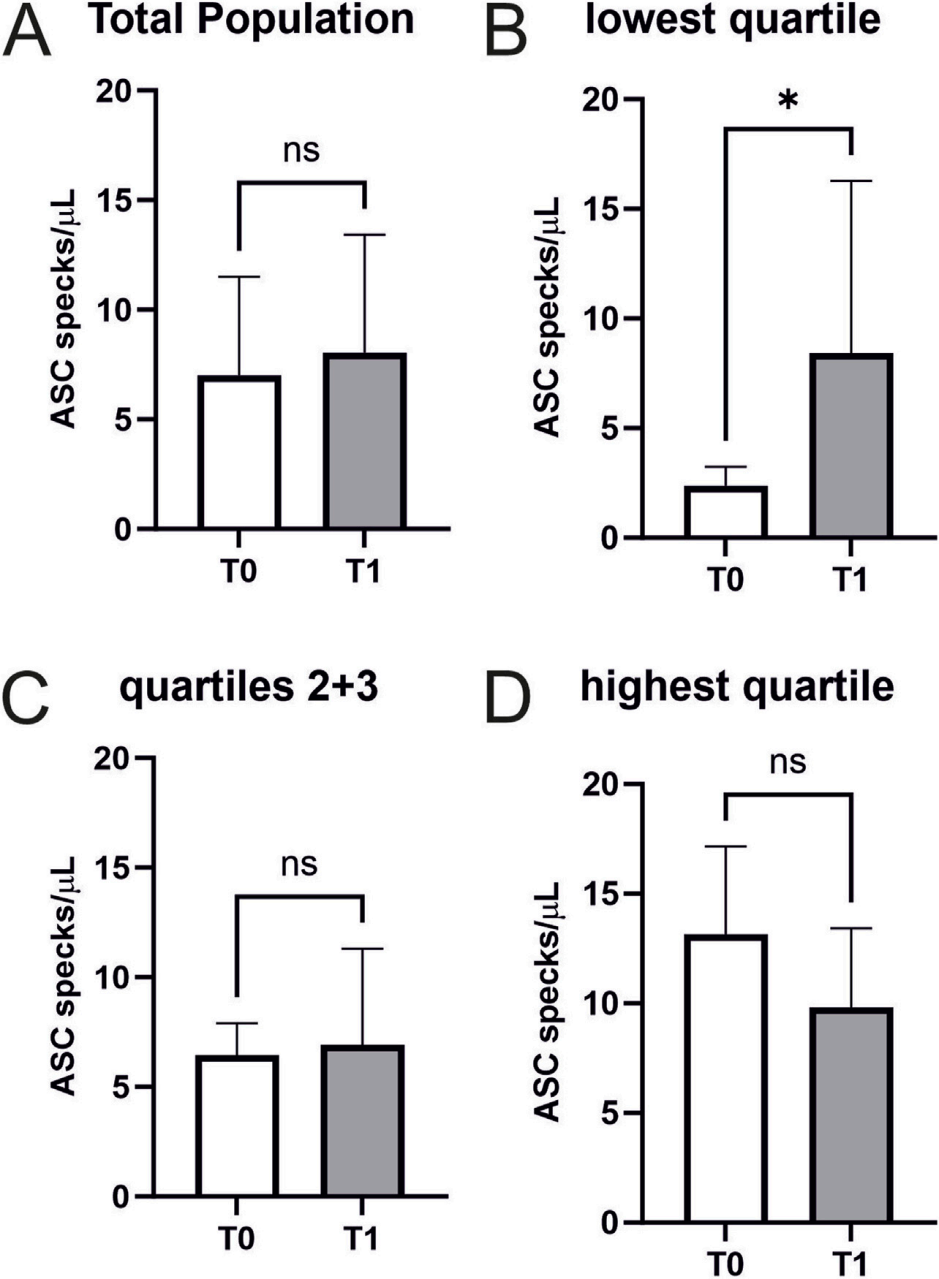
Extracellular ASC specks increase in individuals with low starting values in response to maximum exercise **(A)** Extracellular ASC specks were measured using flow cytometry. Samples were collected immediately before CPET (T0) and immediately after CPET (T1) (N = 45). Absolute values of ASC specks per microliter serum are depicted. **(B–D)** Subgroup analysis of the quartile with the lowest ASC speck concentration at T0 (n = 12), the middle group (n = 22), and the quartile with the highest ASC speck concentration at T0 (n = 11) were performed. Data are expressed as mean ± standard deviation *p < 0.05. Paired student-t tests were used to analyze the data.

Performing a CPET does not increase ASC speck serum concentrations in cardiovascularly healthy athletes (7.0 ± 4.5 vs. 8.0 ± 5.4 ASC specks/µl, *p* = 0.3) (Figure 3A).

An exploratory analysis of quartiles of baseline ASC speck concentrations analogue to a previous analysis in marathon runners revealed that exercise-induced changes differ between atheletes with low baseline values compared to those with high baseline values (lowest quartile n = 12 with 9 females and 3 males, second quartile n = 11 with 6 females and 5 males, third quartile n = 11 with 3 females and 8 males, highest quartile n = 11 with 3 females and 8 males) (Kogel et al., 2022). Individuals with low baseline values showed a significant increase in ASC speck serum concentrations (2.37 ± 0.84 vs. 8.43 ± 7.52 ASC specks/µl, *p* < 0.05) (Figure 3B). We found no significant changes in the other quartiles (Figure 3C/D). Notably, CK (4.2 ± 3.8 vs. 4.3 ± 3.8 mmol/L, *p* < 0.01), CK-MB (0.3 ± 0.1 vs. 0.4 ± 0.1 mmol/L, *p* < 0.05), and creatinine (85.8 ± 14 vs. 88.9 ± 14.6 mmol/L, *p* = 0.001) showed small but significant increases after exercise, while hsTroponin remained unchanged (6.6 ± 2.3 vs. 6.4 ± 2.0 pg/mL, *p* = 0.075; Table 2).

**TABLE 2:** Exercise-induced changes of laboratory parameters.

Blood parameter before and after exercise	T0	T1	p
	Mean (SD)	Mean (SD)	
CK, mmol/L	4.2 (3.8)	4.3 (3.8)	**0.002**
CK-MB, mmol/L	0.3 (0.1)	0.4 (0.1)	**0.019**
hsTroponin, pg/mL	6.6 (2.3)	6.4 (2.0)	0.075
NTproBNP, pg/mL	66.3 (32.2)	68.8 (35.1)	0.068
Creatinine, mmol/L	85.8 (14.0)	88.9 (14.6)	**0.001**
ASC specks, 1/µL	7.0 (4.5)	8.0 (5.4)	0.298

Blood samples were taken before (T0) and 15 min after CPET (T1). Student’s paired *t*-test were used. CK, creatine kinase; CK-MB, creatine kinase myocardial band, hs, high sensitive; NTproBNP, N-terminal-pro-brain natriuretic peptide; ASC, apoptosis associated speck-like protein containing a CARD; 1/µL, total number of particles per microlitre.

### Increasing ASC speck concentrations in response to exercise are associated with lower cardiopulmonary capacity

**FIGURE 4:**
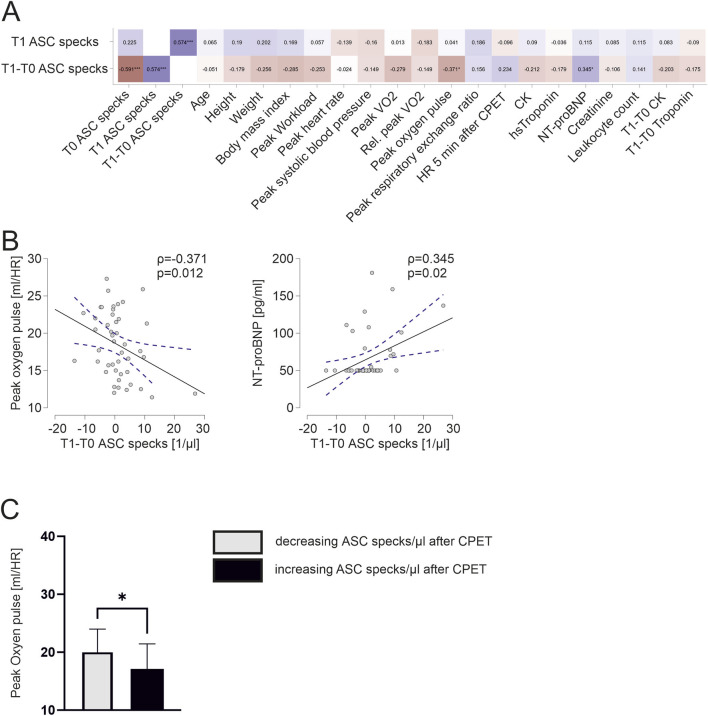
Exercise-induced increases in ASC specks correlate to lower cardiopulmonary capacity and athletes with increasing ASC specks have a significantly lower peak oxygen pulse **(A)** Spearman correlation matrix of extracellular ASC specks after CPET and exercise-induced changes with clinical parameters, parameters of CPET and blood parameters. **(B)** Exemplary scatter plots and linear regression (95% confidence interval in blue) of correlations of extracellular ASC specks. The Spearman correlation coefficient rho (ρ) was used to analyze the data. **(C)** Subgroup comparison of those with an decrease of ASC speck concentration vs. those with an increase of ASC speck concentration in response to maximum exercise. Data are expressed as mean ± standard deviation *p < 0.05,**p < 0.01,***p < 0.001. Student`s t-tests were used to analyze the data.

We found a significant negative correlation of the exercise-induced change of ASC speck concentration to peak oxygen pulse (ρ = −0.371, *p* = 0.012). Interestingly, an increase in ASC speck concentrations in response to maximum exercise significantly correlates to higher NT-proBNP values at baseline (ρ = 0.345, *p* = 0.02; Figure 4A, B). Athletes with an increase in ASC speck serum concentrations in response to maximum exercise had a significant lower peak oxygen pulse compared with athletes with decreasing ASC speck concentrations (17.1 ± 4.2 vs. 19.8 ± 4.1, *p* < 0.05; Figure 4C).

### Interleukin 18 but not interleukin 1β increases after exercise

**FIGURE 5:**
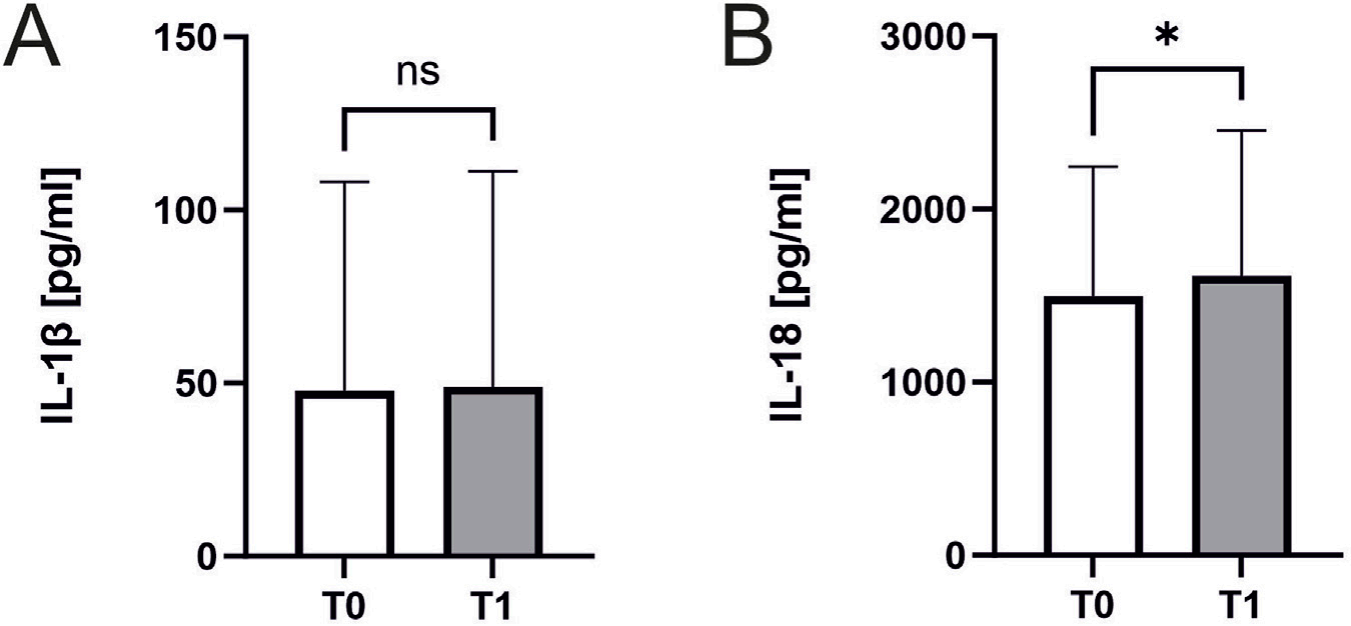
Interleukin 18 and Interleukin 1β concentrations before and after exercise. Effect of exercise on interleukin levels. Samples were collected immediately before CPET (T0) and immediately after CPET (T1). Levels of **(A)** IL-1β (n = 23) and **(B)** IL-18 (n = 45 before and n = 44 after CPET) in human serum of athletes. Data are expressed as mean ± standard deviation*p < 0.05. Paired student-t tests were used to analyze the data.

Interleukin 18 significantly increased in response to exercise (1496 ± 741 pg/mL, n = 45 vs. 1613 ± 832 ng/mL, n = 44; *p* = 0.02; Figure 5A). In contrast Interleukin 1β remained unchanged (48 ± 59 pg/mL vs. 49 ± 61 ng/mL, n = 23 *p* = 0.55, Figure 5B). There were no significant correlations of Interleukin concentrations to ASC specks or parameters of cardiopulmonary capacity.

## Discussion

The main findings of the study are that low ASC speck baseline values as well as an increase in response to exercise are associated with lower individual cardiopulmonary capacity measured by peak oxygen pulse and peak workload in healthy athletes. Athletes with lowest baseline ASC speck concentrations have a lower BMI, higher resting heart, lower peak VO2 and lower peak oxygen pulse. Overall, ASC speck concentrations showed no significant change after CPET. However, athletes with an increase in ASC speck concentrations in response to CPET were characterized by a lower peak oxygen pulse.

While changes in blood parameters, especially inflammatory parameters in response to exercise are well studied, blood parameters to assess or predict cardiovascular capacity are missing (Ibrahim et al., 2023). ASC specks at baseline significantly correlate to BMI, which in athletes can be assumed to be mainly a difference in muscle mass. Additionally, we found a positive correlation to parameters of cardiovascular capacity such as peak VO2 and peak oxygen pulse. Both have been shown to be correlated to a decrease in mortality (Lavie et al., 2004; Myers et al., 2002).

In contrast to our previous findings in marathon runners (Kogel et al., 2022),we did not find an overall increase in ASC specks in response to maximum exercise during CPET. The difference between running and cycling on a semi-recumbent ergometer does not explain the differences between marathon and CPET, since longer bouts of cycling also induce inflammasome activation (Nieman et al., 2020). CPET differs from marathon in several aspects including in duration while both induce near maximal exhaustion. Several studies reported an increase of hsTroponin in response to excessive bouts of exercise such as marathons and ultra-marathons (Shave et al., 2002; Shave et al., 2007; Fortescue et al., 2007). In our study, we found no increase of hsTroponin in response to CPET in the healthy athletes indicating no relevant cardiac damage. Interestingly, athletes in the lowest quartile of ASC speck baseline values exhibit a significant increase in ASC speck concentrations in response to marathon as well as in response to CPET. Athletes in the quartiles with higher ASC speck concentrations show no exercise-induced regulation in response to CPET, which was different to our findings in marathon runners (Kogel et al., 2022). The exercise-induced change in ASC specks is correlated to body composition and peak oxygen pulse albeit to a lesser degree than baseline values. An increase in ASC specks after exercise is significantly correlated to higher NT-proBNP values, although all baseline values were in the normal range.

Of note, no athlete in our study had increased values of CRP. Since Inflammasome activation has been described in a wide variety of disease, pre-existing inflammation of other sources could possibly conceal exercise-induced regulation of ASC speck release (Franklin et al., 2014; Basiorka et al., 2018; Martínez-García et al., 2019).

There are limitations to our study. Our population included only cardiovascularly healthy and young athletes, therefore transferring the data to older populations or populations with chronic diseases is not possible. Measurements of blood parameters were performed in human serum and plasma. Other tissues are not available. Any novel observation warrants confirmation in additional cohorts. The study was purely observational and we were not able to explore a possible underlying molecular mechanism. Regarding correlation analysis the sample size is small and correlation is only modest, nevertheless significant. In our case, many IL-*1β* concentrations in particular were below the technical detection limit. Temporally, interleukin release follows activation of the inflammasome, therefore relevant increases may occur in the hours after CPET, and blood samples from this time were not available in this study.

In conclusion, there is no significant increase of ASC specks in healthy athletes in response to a short intensive exercise bout. Additionally, our data suggests that low ASC speck baseline values and an increase in response to CPET are associated with low cardiopulmonary capacity measured by peak oxygen pulse and peak workload. Further studies should evaluate whether exercise training results in higher baseline ASC speck values either by improving cardiopulmonary capacity or muscle mass. These data provide the rational to further test a potential use of serum ASC specks to predict and guide individual response to physical training and to help assess individual cardiopulmonary capacity.

## Data Availability

The raw data supporting the conclusions of this article will be made available by the authors, without undue reservation.

## Supplementary material

SUPPLEMENTARY FIGURE S1 Exemplary dot blots of flow cytometric measurements of ASC specks. Depicted are dot plots of human serum which are labeled with ASC-B3-Alexa-647 antibodies (stained) or without antibodies(unstained) in the 640-nm channel (APC). Before CPET (T0) and 15 min after CPET (T1).
