# Supplementary figures and images for: ASC speck serum concentrations, a component of sterile cellular inflammation, are associated with individual cardiopulmonary capacity
Source: Front Physiol. 2024 Oct 1;15:1394340. doi: 10.3389/fphys.2024.1394340 (PMC11473406; doi:10.3389/fphys.2024.1394340)

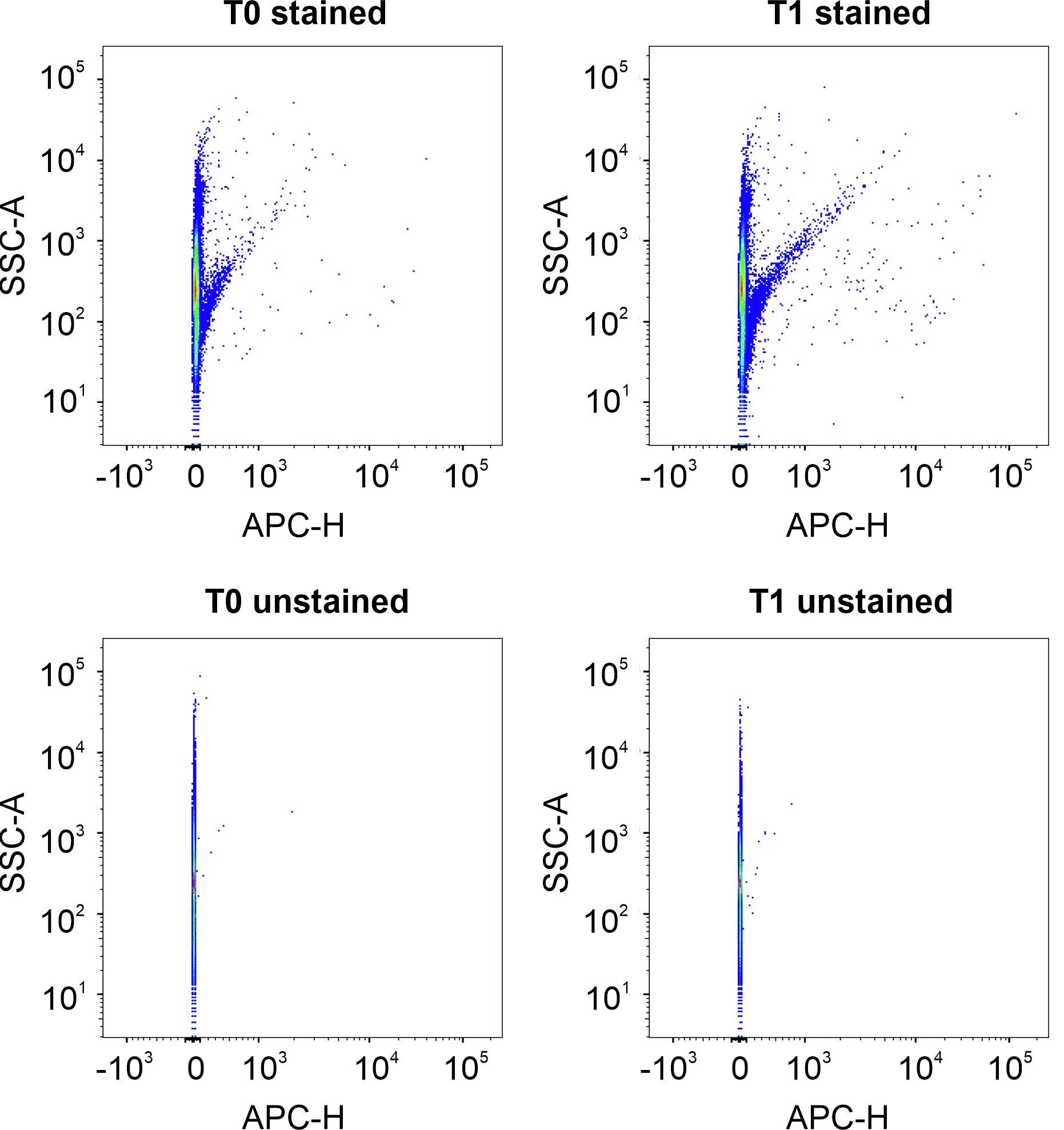

Supplement: Supplementary file 1 [file Image1.JPEG]
